# Management of Cardiovascular Complications in Antiphospholipid Syndrome: A Narrative Review with a Focus on Older Adults

**DOI:** 10.3390/jcm13113064

**Published:** 2024-05-23

**Authors:** Marco Bernardi, Luigi Spadafora, Silvia Andaloro, Alessandra Piscitelli, Giovanni Fornaci, Chiara Intonti, Alberto Emanuele Fratta, Chieh-En Hsu, Karol Kaziròd-Wolski, Theodora Metsovitis, Giuseppe Biondi-Zoccai, Pierre Sabouret, Emanuele Marzetti, Stefano Cacciatore

**Affiliations:** 1 Department of Clinical, Internal Medicine, Anesthesiology and Cardiovascular Sciences, Sapienza University of Rome, Piazzale A. Moro 5, 00185 Rome, Italy; luigispadafora167@gmail.com (L.S.); giovanni.fornaci@uniroma1.it (G.F.); chiaraintonti94@gmail.com (C.I.); 2 Department of Translational Medicine and Surgery, Università Cattolica del Sacro Cuore, Largo F. Vito 1, 00168 Rome, Italy; silvia.andaloro01@icatt.it; 3 Faculty of Medicine and Surgery, Università Cattolica del Sacro Cuore, Largo F. Vito 1, 00168 Rome, Italy; alessandra.piscitelli01@icatt.it (A.P.); albertoemanuele.fratta02@icatt.it (A.E.F.); chiehen.hsu01@icatt.it (C.-E.H.); theodora.metsovitis01@icatt.it (T.M.); 4 Institute of Medical Sciences, Collegium Medicum, Jan Kochanowski University, Al. IX Wieków Kielc 19A, 25-317 Kielce, Poland; kk-wolski@wp.eu; 5 Department of Medical-Surgical Sciences and Biotechnologies, Sapienza University of Rome, Via XXIV Maggio 7, 04100 Latina, Italy; giuseppe.biondizoccai@uniroma1.it; 6 Cardiology Unit, Santa Maria Goretti Hospital, Via L. Scaravelli, 04100 Latina, Italy; 7 Heart Institute, Pitié-Salpétrière Hospital, Sorbonne University, 47-83 Bd. de l’Hôpital, 75013 Paris, France; cardiology.sabouret@gmail.com; 8 National College of French Cardiologists, 13 Niepce, 75014 Paris, France; 9 Department of Geriatrics, Orthopedics, and Rheumatology, Università Cattolica del Sacro Cuore, Largo F. Vito 1, 00168 Rome, Italy; emanuele.marzetti@policlinicogemelli.it; 10 Fondazione Policlinico Universitario “A. Gemelli” IRCCS, Largo A. Gemelli 8, 00168 Rome, Italy

**Keywords:** stroke, thrombosis, lupus anticoagulant, anti-β2-glycoprotein I antibodies, anti-cardiolipin antibodies, monoclonal antibodies, miRNA

## Abstract

Antiphospholipid syndrome (APS), also known as Hughes syndrome, is an acquired autoimmune and procoagulant condition that predisposes individuals to recurrent thrombotic events and obstetric complications. Central is the role of three types of antiphospholipid antibodies that target phospholipid-binding proteins: lupus anticoagulant (LAC), anti-β2-glycoprotein I (β2-GPI-Ab), and anti-cardiolipin (aCL). Together with clinical data, these antibodies are the diagnostic standard. However, the diagnosis of APS in older adults may be challenging and, in the diagnostic workup of thromboembolic complications, it is an underestimated etiology. The therapeutic management of APS requires distinguishing two groups with differential risks of thromboembolic complications. The standard therapy is based on low-dose aspirin in the low-risk group and vitamin K antagonists in the high-risk group. The value of direct oral anticoagulants is currently controversial. The potential role of monoclonal antibodies is investigated. For example, rituximab is currently recommended in catastrophic antiphospholipid antibody syndrome. Research is ongoing on other monoclonal antibodies, such as daratumumab and obinutuzumab. This narrative review illustrates the pathophysiological mechanisms of APS, with a particular emphasis on cardiovascular complications and their impact in older adults. This article also highlights advancements in the diagnosis, risk stratification, and management of APS.

## 1. Introduction

Antiphospholipid syndrome (APS), also known as Hughes syndrome, is a multifaceted autoimmune disorder characterized by the presence of a procoagulant state that predisposes individuals to recurrent thrombotic events and obstetric complications. Central to the pathogenesis of APS are three types of antiphospholipid antibodies (aPLs): lupus anticoagulant (LAC), anti-β2-glycoprotein I (β2-GPI-Ab), and anti-cardiolipin (aCL) [1]. These antibodies specifically target proteins that bind to phospholipids and act on endothelial cells to increase platelet adhesion and influence the balance of coagulation and anticoagulation molecules. This action results in a hypercoagulable state, leading to intravascular thrombosis and subsequent multiorgan damage, including harm to the cardiovascular system [2].

The estimated incidence of APS is 1–2 cases per 100,000 individuals, with a prevalence ranging from 40 to 50 cases per 100,000. Among patients with pregnancy disorders, the prevalence of thrombotic events, whether arterial or venous, is approximately 9–10%, and the detection of antiphospholipid antibodies in lab tests is around 6–9%. Over the past few decades, the mortality rate has decreased significantly from 50 to 80% [3,4] to around 20% [5]. This improvement is primarily due to advances in therapy, particularly the use of anticoagulants, corticosteroids, plasma exchanges, and intravenous immunoglobulins [5].

APS can be classified into two clinical subtypes: primary and secondary. Primary APS involves the detection of antiphospholipid antibodies without any other systemic diseases [6]. Secondary APS, on the other hand, is associated with other disorders, with systemic lupus erythematosus (SLE) being the most common, followed by other autoimmune diseases like Sjögren’s syndrome, rheumatoid arthritis, and vasculitis as well as solid and hematological malignancies, infections, and more. About half of patients with SLE exhibit clinical manifestations of APS despite the presence of antiphospholipid antibodies [6]. In 2023, the American College of Rheumatology/European League Against Rheumatism (ACR/EULAR) updated the “Sapporo—2006 revised criteria” (or Sydney criteria) [1] with new diagnostic tools that encompass both clinical and laboratory domains, improving specificity and providing better diagnostic accuracy [6].

The aim of this review is to illustrate the pathophysiological mechanisms of APS, with a particular emphasis on cardiovascular complications and their impact in older adults. This review highlights diagnostic methods and discusses advancements in the risk stratification and management of APS.

## 2. Cardiovascular Complications of Antiphospholipid Syndrome: Why Does It Matter?

Recurrent thromboses, primarily venous and often in the lower extremities, are a prominent feature of APS. However, thromboses can also occur in other sites, including the pelvic, pulmonary, hepatic, portal, renal, and inferior vena cava veins, and sometimes even in the superficial venous circulation [7]. Arterial thromboses are most frequently observed in the cerebral circulation, leading to strokes or transient ischemic attacks. Other arteries, such retinal, coronary, renal, and mesenteric arteries, can also be involved [8]. Other relatively common signs of APS include thrombocytopenia, autoimmune hemolytic anemia, microangiopathic syndrome, pulmonary hypertension, and livedo reticularis [9]. A rare but severe manifestation, known as catastrophic antiphospholipid antibody syndrome (CAPS), may result in multiorgan failure [10].

**Figure 1:**
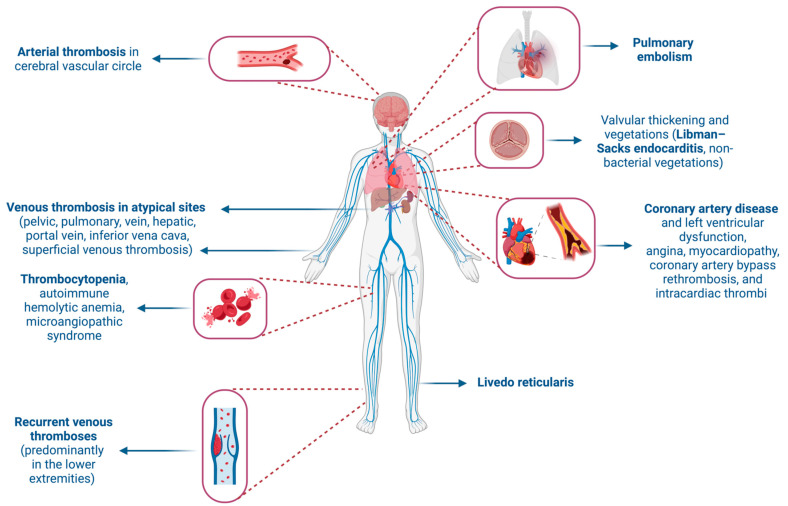
Clinical manifestations of antiphospholipid syndrome. Created with BioRender.com (accessed on 2 May 2024).

Cardiovascular manifestations are prominent in APS, with valvular disease being prevalent. This includes thickening and non-bacterial valvular vegetations (Libman–Sacks endocarditis) affecting about 30% of patients and significantly increasing stroke risk [9,11]. The mitral and aortic valves are most commonly affected [12]. Moreover, the available literature on heart valve surgery and prosthetic valve management in APS indicates that there is a heightened risk of morbidity and mortality, both in the short and long term [13,14,15,16]. Patients with APS also face a higher risk of coronary artery disease and left ventricular dysfunction, pulmonary thromboembolism, microvascular thrombosis, and the rupture of atherosclerotic plaques [17,18]. The risks of valvular involvement and coronary artery disease are heightened in the presence of aCL and LAC [19,20]. The cardiac complications of APS can manifest as angina, cardiomyopathy, graft thrombosis after coronary artery bypass surgery, and intracardiac thrombi, emphasizing the broad impact of this syndrome on cardiovascular health [9] (Figure 1).

## 3. Pathophysiology of Antiphospholipid Syndrome with a Focus on the Cardiovascular System

aPLs not only act as indicators for APS but also play a vital role in its development and progression. Studies in mice demonstrated that administering aPLs induced manifestations resembling those of APS, indicating that the presence of aPLs is necessary but not sufficient to cause blood clot formation [21,22]. This has led to the hypothesis of a “two-hit” pathophysiological mechanism [2]. The first hit entails the presence of aPLs, while the second involves disturbances in endothelial homeostasis, potentially triggered by factors like pregnancy, infections, and surgery [2]. This theory is supported by findings in CAPS, where a “second hit” is detectable in up to 80% of cases [23].

The prothrombotic effects of aPLs stem from their ability to disrupt the balance between prothrombotic and anticoagulant systems, affecting various cells and pathways such as endothelial cells, platelets, monocytes, neutrophils, the complement system, fibrinolysis, and coagulation pathways. Endothelial cells play a pivotal role in the pathophysiology of APS. In vitro studies highlighted the importance of the activation of Apo E receptor 2 (Apoer2), which in turn triggers the nuclear factor kB (NF-kB) and P38 pathways [24,25,26,27]. Following this activation sequence, a downregulation of anti-inflammatory and vascular protective genes occurs together with enhanced interaction between immune cells and the endothelial layer, a reduction in the local production of nitric oxide leading to vasoconstriction, and the release of tissue factor [24,25,26,27]. Platelets have a dual role in APS. They are well known for their role in thrombus formation, but they also interact with lymphocytes, forming circulating complexes in patients with APS [27]. This interaction fosters a proinflammatory state and continuous mild platelet activation, predisposing to thrombosis, particularly in the presence of a second hit [2]. In monocytes, aPLs induce the overexpression of tissue factor and proinflammatory cytokines such as tumor necrosis factor alpha (TNF-α) and interleukin 1-beta (IL-1β), enhancing the prothrombotic environment [28].

The complement system also plays a critical role in APS. Evidence suggests the presence of a persistent, mild activation of the complement system in patients with APS, reinforcing the link between inflammation and coagulation [29]. This is particularly supported by studies in animal models [30,31]. Moreover, aPLs have been shown to inhibit fibrinolysis, potentially through a direct action on fibrinolytic factors, further contributing to the thrombotic imbalance in APS [32]. All these mechanisms contribute to the pathophysiology of cardiovascular complications in APS, including valvular diseases, atherosclerosis, myocardial ischemia, pulmonary thromboembolism, hypertension, myocardial dysfunction, and rare cases of intracavitary thrombosis. Immunoglobulin deposits, especially IgG, on valve leaflets can initiate an inflammatory process leading to the formation of sterile vegetations (Libman–Sacks endocarditis), evolving into fibrosis and valvular deformation [33].

The interaction between aPLs and the endothelium not only fosters a proinflammatory and procoagulant state conducive to atherosclerosis but also involves an indirect mechanism through autoantibody cross-reaction by oxidative stress [34]. Complexes formed between β2-GPI-Ab and oxidized low-density lipoproteins may contribute to chronic vascular inflammation, affecting nitric oxide generation, monocyte adhesion, vasodilation inhibition, and local oxidative stress [34,35]. This proatherogenic environment, coupled with prothrombotic activity, can precipitate cardiac ischemia, with traditional risk factors exacerbating this risk [36]. The prevalence of myocardial infarction (usually ST-elevation myocardial infarction) in patients with APS is 1.2% and is further elevated in those with concurrent SLE (3.8%), underlining the significant contribution of thromboembolic activity in acute coronary syndromes in APS [34]. A number of studies have highlighted the association between APS and myocardial infarction with non-obstructive coronary arteries (MINOCA) [37,38,39,40,41], with APS that may be diagnosed in up to 15.5% of patients with MINOCA [42].

Pulmonary arterial hypertension (PH) is a relatively frequent complication of APS with a prevalence ranging between 1.8 and 3.5%. PH is frequently induced by pulmonary embolism (usually related to deep vein thrombosis of the lower limbs), defined as chronic thromboembolic pulmonary hypertension (CTEPH), the most common form of PH in APS. aPLs could lead to PH by promoting remodeling of pulmonary vessel cells through endothelin-1, thereby causing precapillary pulmonary hypertension [43,44].

## 4. Diagnosis of Antiphospholipid Syndrome with Cardiovascular Manifestations

A side-to-side comparison of the main diagnostic criteria established for APS can be found in Table 1. Similar to the Sydney criteria, the updated APS classification criteria released by ACR/EULAR are not designed to be diagnostic, but are often used to identify patients with homogeneous characteristics who can be included in clinical studies on APS [6]. These criteria consist of a primary inclusion criterion, which requires at least one positive assay of aPLs within three years of detection of a clinical criterion, in addition to other criteria grouped into six clinical domains and two laboratory domains. The six clinical domains are (1) macrovascular venous thromboembolism (VTE), (2) macrovascular arterial thrombosis, (3) microvascular presentations (skin, lung, renal, myocardial, and adrenal), (4) obstetric pathology (such as fetal death, preeclampsia), (5) cardiac valve involvement (thickening or vegetation), and (6) unexplained thrombocytopenia. The two laboratory domains are (1) LAC positivity and (2) aCL and β2-GPI-Ab positivity; the antibody titers of the latter must be determined with a standardized ELISA test. Each criterion is assigned a score from one to seven, and to diagnose APS, it is necessary to reach at least three points from each domain (clinical and laboratory).

ACR/EULAR criteria, in their dual role of diagnosis and classification, categorize patients into risk classes, thereby aiding in the clinical management of these patients. Accordingly, individuals with APS are classified into low- and high-risk groups. Low-risk individuals, also called asymptomatic aPL carriers, may be identified during diagnostic investigations for rheumatologic disease, in-depth analysis of recurrent miscarriages, unexplained prolongation of activated partial thromboplastin time (aPTT), or in the absence of any episode of thrombosis (arterial or venous) or obstetric APS [6]. This group has a marginally increased risk of thrombosis compared with the general population, estimated as 1.3% per year according to a study by Pengo et al. [45]. High-risk individuals are those with a history of thrombotic episodes (arterial or venous) or obstetric APS. They often have an antibody profile characterized LAC positivity (confirmed in two or more measurements at least twelve weeks apart), multiple aPL positivity (double or triple), and persistently high aPL titers. This group has an estimated 37% incidence of thromboembolic complications over 10 years [45,46].

Diagnosing APS with only cardiovascular involvement is particularly challenging, as it first necessitates ruling out other more common and potential secondary causes of cardiovascular damage. Valvular involvement is the most common cardiac manifestation of APS, and it is defined as the coexistence of aPLs along with the echocardiographic (transthoracic or transesophageal) identification of a valve lesion and/or moderate to severe regurgitation and/or stenosis of the mitral or aortic valve. Valve vegetation is characterized by leaflet thickness greater than 3 mm in the proximal or middle part of the cusps, or the presence of irregular nodules on the atrial area of the mitral valve or the vascular side of the aortic valve. As mentioned, other potential causes of valvular degeneration, such as a history of rheumatic disease or infective endocarditis, must be excluded [1].

Intracardiac thrombosis is one of the possible manifestations of APS. In the general population, it is more frequently associated with reduced left ventricular ejection fraction, cardiac wall aneurysms, and akinesia of certain high-risk segments such as the cardiac apex. The simultaneous involvement of two or three cardiac chambers, in the absence of the anatomical context mentioned above, diagnosed by transthoracic or transesophageal echocardiogram and confirmed at cardiac magnetic resonance imaging or autopsy examination, could suggest a direct role of autoantibodies (mainly aCL IgG), independent of ventricular dysfunction [47,48]. A study found early diastolic changes and high left ventricular filling pressures in young patients with APS, without other cardiovascular risk factors, who subsequently developed heart failure [49]. Further studies are needed to assess whether echocardiogram should be used as an early screening tool for myocardial involvement in APS. Another study correlated the presence of a high blood titer of aCL IgM with manifest heart failure [47]. More research is needed to establish whether high aCL antibody titers can serve as a predictive marker for heart failure in APS.

In conclusion, for younger patients with cardiovascular diseases, more common secondary causes must be excluded first; once ruled out, these patients should be screened for and monitored according to EULAR criteria for APS over a three-year period, along with other causes of thrombophilia. A high thrombotic burden, even in the presence of other more plausible secondary causes, may indicate the coexistence of APS.

## 5. Management Strategies for Antiphospholipid Syndrome with Cardiovascular Complications

### 5.1. Current Recommendations for the Management of Antiphospholipid Syndrome

General management measures for APS, regardless of risk classification, include controlling cardiovascular risk factors such as hypertension, dyslipidemia, diabetes, smoking, and physical inactivity. Additionally, regular screening including Doppler ultrasounds is recommended for the early detection of complications. In circumstances such as immobilization, hospitalization, surgery, and puerperium, anticoagulation therapy is necessary, even in cases considered to be low-risk. Measures to be implemented also include patient education on adherence to therapy, discontinuation of oral contraceptives or postmenopausal hormone therapy, and intensification of monitoring during pregnancy or the postpartum period.

**Figure 2:**
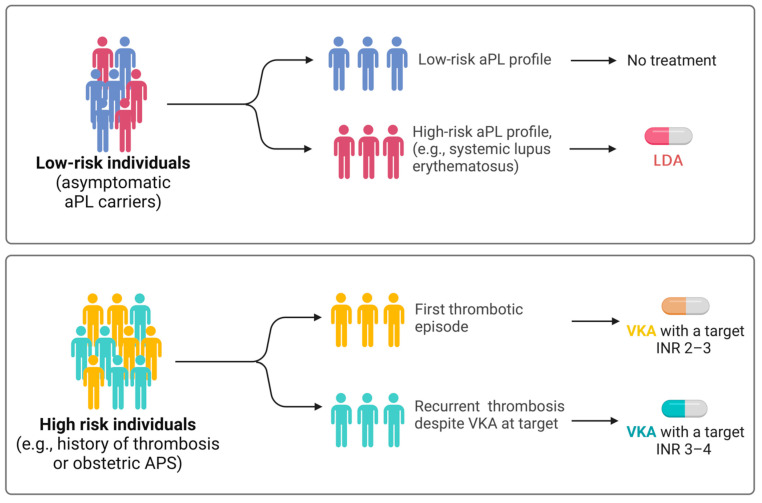
Recommended treatment approaches according to the latest recommendations by the European League Against Rheumatism (EULAR). In case of recurrent thrombosis despite anticoagulant therapy in the therapeutic range, it is recommended to increase the INR in the 3–4 range. Abbreviations: APS, antiphospholipid syndrome; INR, international normalized ratio; VKA, vitamin K antagonist. Created with BioRender.com (accessed on 2 May 2024).

In low-risk individuals (asymptomatic carriers of aPLs who do not fulfill any criteria for classification of vascular or obstetric APS) with a high-risk aPL profile, such as those diagnosed with SLE [50,51], prophylactic treatment with low-dose aspirin (LDA) (75–100 mg daily) is recommended (level of evidence 2a) (Figure 2) [6].

LDA treatment may also be considered in individuals with a low-risk aPL profile (level of evidence 2b). A meta-analysis of observational studies involving a total of 497 patients with isolated antiphospholipid antibody positivity demonstrated a reduced risk of thromboembolism in patients treated with LDA (adjusted hazard ratio: 0.43, 95% confidence interval: 0.25–0.75) [52]. In women who are not pregnant but have had a previous episode of obstetric APS (with or without a diagnosis of SLE), treatment with LDA as prophylaxis is recommended (level of evidence 2a).

### 5.2. Role of Anticoagulant Therapy and Related Challenges

In high-risk individuals, such as those experiencing their first episode of deep vein thrombosis, treatment with vitamin K antagonists (VKAs) targeting an INR of 2 to 3 is mandatory (level of evidence 1b) (Figure 2). If the episode of venous thrombosis is unprovoked, anticoagulant therapy should be extended long-term (level of evidence 2b). A retrospective study conducted by Rosove et al. [53] has shown that recurrent thromboembolism occurs at a rate of 30% annually in individuals with APS who have discontinued anticoagulant therapy. If the episode of venous thrombosis is provoked (i.e., attributable to immobilization, surgery, concomitant estrogen–progestin contraceptive, or postmenopausal therapy), anticoagulant treatment should be continued according to international guidelines for VTE for a duration equal to that recommended for patients without APS (level of evidence 5). Extending anticoagulant therapy may be considered if other risk factors coexist. Some studies have investigated, often with conflicting results, the use of direct oral anticoagulants (DOACs) if contraindications to VKAs are present (allergies, intolerances) or when a target INR cannot be achieved despite adherence to therapy [54,55]. A randomized clinical trial was conducted to determine whether rivaroxaban was noninferior to VKAs for preventing recurrent thrombosis in 190 patients with APS and arterial or venous thrombosis [56]. There were more episodes of recurrent thrombosis in the rivaroxaban-treated group (6.3% of VKA-treated participants versus 11.6% of those receiving rivaroxaban; risk ratio: 1.83; 95% confidence interval: 0.71–4.76), with comparable bleeding risk.

A systematic review with a comparative analysis of the international guidelines by Pastori et al. [57] addressed that while VKAs are the main treatment for patients with arterial thrombosis or triple aPL positivity, DOACs may be preferred in patients with venous thrombosis and single or double aPL positivity. Another systematic review with meta-analysis demonstrated that dabigatran or apixaban yielded a similar rate of recurrent thrombosis and major bleedings to VKAs, while rivaroxaban was associated with a higher risk of recurrent arterial thromboses [58]. A more recent study by Bakow et al. [59] found that the incidence rate of recurrent venous thrombosis in APS patients with single aPL positivity was comparable to that of patients without APS who had previously experienced venous thrombosis and did not vary depending on the anticoagulation regimen (DOACs or VKAs), suggesting that single-positive APS patients may not require extended anticoagulation with VKAs. Therefore, although the guidelines for the management of APS strictly recommend the use of VKAs, evidence suggests that alternative regimens may be considered in patients with APS with an estimated low risk of recurrent VTE to overcome the contraindications linked with VKA administration. Nevertheless, there is the need to conduct further clinical trials in order to explore the efficacy and safety profiles of DOACs or discontinuation of anticoagulation in low-risk aPL syndrome.

There is evidence on the challenges associated with INR fluctuations in patients with concomitant APS and a mechanical valve [60,61]. The current guidelines recommend targeting the INR based on both patient-related risk factors and the thrombogenicity of the mechanical valve. Furthermore, they advise considering a bioprosthesis in patients at increased risk of thromboembolism, including APS [62]. In light of these complexities, a tailored approach to anticoagulation management in APS patients with valvular heart disease is essential. This approach should consider individual risk factors and include vigilant monitoring in order to optimize outcomes and minimize complications.

### 5.3. Specific Considerations for Managing Cardiovascular Complications

In the event of a first episode of arterial thrombosis (such as ischemic stroke or myocardial infarction), treatment with VKA targeting an international normalized ratio (INR) of two to three is recommended [39] (Figure 2). The currently available evidence suggests that regular-dose aspirin (325 mg) or dual antiplatelet therapy may have equivalent efficacy to VKAs in the treatment of a first episode of arterial thrombosis; however, the results must be interpreted in light of significant limitations in the available evidence [63].

The use of DOACs is contraindicated in patients with triple aPL positivity and arterial events, as it is associated with a high risk of recurrence (level of evidence 1b) [6].

In patients with APS and recurrent episodes of deep vein thrombosis or arterial thrombosis despite VKA treatment, after ensuring proper adherence to prescribed therapy and maintenance of INR at target, the addition of LDA (level of evidence 4d) and the increase in the INR target to 3–4 or switching to low-molecular-weight heparin (LMHW) may be considered (Figure 2). A systematic review based on retrospective studies observed a reduction in thrombotic events if the INR target was maintained above three (3.8%; 4% arterial and 1% venous thrombosis) compared with an INR below three (23%; 13% arterial and 16% venous thrombosis) [64].

Although definitive studies on this topic are lacking, aPL-positive patients with valve involvement should receive LDA. If the risk of embolization increases (e.g., valve vegetations) or in case of myocardial infarction, anticoagulation should be considered. In individuals with APS-related valvular involvement, valve lesions do not recede with antiplatelet therapy and VKA. However, this treatment regimen appears to prevent the occurrence of embolic events [65]. There are no clear recommendations for patients who, despite anticoagulant therapy, have a thromboembolic event secondary to cardiac vegetations. The use of alternative therapies, such as immunomodulatory agents [66] including rituximab for hematologic manifestations (e.g., thrombocytopenia) [67] and hydroxychloroquine and eculizumab in refractory APS [68], has been explored. However, further research is warranted to establish their efficacy and safety.

## 6. Cardiovascular Implications of Antiphospholipid Syndrome in Older Adults

### 6.1. Clinical Manifestations of Antiphospholipid Syndrome in Older Adults

APS in advanced age poses a significant concern owing to frequent atypical presentations and a lack of evidence on the management of APS in older adults [69]. Notably, older patients are diagnosed with single-positive APS more often than their younger counterparts (82.8% vs. 59.8%) [70], presenting more frequently with primary APS (72.7% vs. 53.1%) [71]. Studies indicate a peak in APS incidence late in life, particularly in individuals over 55 years, with a higher prevalence in those older than 75 [72,73]. Contrary to young adults, in old age, the male sex prevails in terms of prevalence (46.6% vs. 18.6%) [9,70,74] and is associated with an increased risk of cardiovascular complications [75,76].

Late-onset APS has a peculiar disease profile [77]. Older adults are more prone to myocardial infarction [70], stroke, and pulmonary embolism [71] compared with younger individuals. The frequency of lower-limb deep vein thrombosis has been reported to be higher in one study [70] and lower in another investigation [71] relative to younger adults. Thrombotic relapse-free survival is shortened in older adults with APS, which is associated with reduced overall survival, possibly due to recurrent arterial and venous thrombosis events [70].

The presence of aPLs in older adults may increase the risk of cardiovascular events [78,79]. However, mixed findings have been reported, with studies showing a link between aCL and dementia and stroke [80,81], while others suggest that aCL and β2-GPI-Ab may be incidental findings in the older population [82,83,84]. Indeed, the prevalence of aPL positivity increases with age, both in healthy individuals [74] and in those with a variety of conditions (e.g., rheumatoid factor positivity [85,86], advanced renal or hepatic failure [87,88], giant-cell arteritis [89], and cancer [81,90]). However, aPL IgG with a titer above 40 units has been shown to be an independent risk factor for thrombosis [81]. LAC is a significant biological marker of APS in older adults, with a higher prevalence in those over 65 years [74], and it is often implicated in vascular events related to APS in this age group [84]. Malignancies, whose incidence increases with aging [91], pose special challenges due to their potential presentations as venous thrombosis [92] and aPL positivity. Hence, an underlying malignancy should be considered in older adults with thrombosis and high aPL titers [69,93,94].

### 6.2. Special Considerations for the Treatment of Antiphospholipid Syndrome in Older Adults

The management of APS in older adults requires special attention owing to their distinct disease profile and treatment-related risks. Efforts should be made to achieve an optimal control of modifiable cardiovascular risk factors. LDA is recommended by EULAR for primary thromboprophylaxis in asymptomatic aPL individuals with high-risk profiles regardless of age [6]. However, special caution is needed in older adults in whom primary prevention with LDA is associated with a high risk of major bleeding and questionable benefits [95]. Anticoagulation with VKAs is the mainstay therapy for cardiovascular complications of APS in older adults [96]. Given the increased bleeding risk in old patients on long-term anticoagulation [97,98], targeting an INR of two to three is recommended in this age group [99]. In individuals with a high thrombotic risk profile who require a higher target INR, a close monitoring of bleeding risk as well as cardiovascular risk factors is necessary [71].

## 7. Future Perspectives in Antiphospholipid Syndrome Research and Management

### 7.1. Emerging Biomarkers

Several autoantibodies, including anti-β2 GPI domain I [100], antiphosphatidylserine/prothrombin [101], antilysobisphosphatidic/endothelial protein C receptor antibodies [102], anti-β2-GPI complexed with HLA class II molecules [103], and antineutrophil extracellular trap antibodies [104], have been proposed as diagnostic markers of APS. However, none of them have a defined role in clinical settings [105]. Some micro-RNAs (miRNAs) have been found to be underexpressed in neutrophils from APS and SLE [106]. Teruel et al. [107] showed that the expressions of miR-19b and miR-20a, two miRNAs belonging to the miR-17-92 cluster that binds directly to tissue factor mRNA and suppresses its translation, were reduced by about 30% in patients with APS and SLE compared with healthy controls. This finding suggests that increased tissue factor expression in the setting of APS may result from altered miRNA signaling. Wu et al. [108] found that treating endothelial cells with β2-GPI-Ab isolated from APS patients induced the secretion of extracellular vesicles with a different miRNA content than those generated after treatment with non-immune IgG. Hence, miRNA alterations can alter the ability of extracellular vesicles exposed to β2-GPI-Ab to activate other endothelial cells. Circulating miRNAs may also serve as biomarkers in obstetric APS [109]. Further evaluation of these miRNAs could enable their use for risk stratification in different clinical manifestations of APS, such as thrombotic events, pregnancy morbidity, and triple aPL positivity. Additionally, miRNAs have the potential to serve as biomarkers for disease progression.

### 7.2. Potential New Treatments and Their Implication in Cardiovascular Complications

Existing prevention and treatment strategies are estimated to fail in approximately 20–30% of obstetric APS and more than 30% of thrombotic APS cases [110]. In recent years, there has been an increase in the understanding of the pathogenesis of APS, which has stimulated research on new targeted therapies [110].

Rituximab, a monoclonal anti-CD20 antibody, is recommended for use as a second-line treatment in APS [111], particularly in cases of refractory CAPS [112]. Potential pitfalls of rituximab include uncertainties regarding its long-term efficacy, adverse effects (e.g., prothrombotic state), and high costs [113]. Furthermore, rituximab may lead to insufficient suppression of aPL-producing plasma cells in patients with APS [114], highlighting the need for combination therapies. In recent years, daratumumab, a monoclonal antibody directed against CD38, has been employed in the management of autoimmune-mediated diseases, including APS [115]. A combination therapy with rituximab to simultaneously target CD20 and CD38 has been tested in preclinical models showing a high degree of synergism [116]. Obinutuzumab, a humanized glycoengineered type II monoclonal antibody targeting CD20, was shown to be more effective than rituximab at inducing B-cell depletion during in vitro whole blood assays, even in the presence of excess B-cell activating factor (BAFF) [117,118]. This suggests that obinutuzumab might serve as a treatment option for APS cases that are resistant to rituximab. Belimumab [119], eculizumab [120,121], and TNF-α blockers such as adalimumab [122,123] have been reported to be effective in some cases, but additional data on their efficacy and safety profiles are needed for their routine clinical use.

## 8. Conclusions

The management of cardiovascular complications in APS, particularly in older adults, remains a significant challenge. In these patients, it is essential to carefully balance the risks and benefits of antiplatelet and anticoagulant therapies due to an increased risk of bleeding. While current treatments primarily include LDA and VKAs, safer and more targeted treatments are highly sought after. Diagnostic approaches are also evolving, with emerging biomarkers like circulating miRNAs proposed to aid in early detection and risk stratification. This is especially relevant in older adults in whom APS may have atypical presentations and lead to severe outcomes.

## Figures and Tables

**Table 1:** Main diagnostic criteria for antiphospholipid syndrome.

Category	Sydney Criteria (2006) [1]	EULAR Recommendations (2023) [6]
Entry criteria	No.	≥1 clinical criterion and ≥1 laboratory criterion.
Diagnosis	≥1 clinical criterion and ≥1 laboratory criterion	Weighted criteria, ≥3 points from clinical domain and ≥3 points for laboratory domain.
Clinical Criteria	Two clinical criteria	Six clinical domains:
	(1) Vascular thrombosis (arterial, venous or small vessel thrombosis). (2) Pregnancy morbidity (one or more unexplained deaths of a morphologically normal fetus at the 10th week of gestation or beyond, or three or more unexplained consecutive spontaneous abortions before the 10th week).	(1) VTE: with (1 point) or without (3 points) high-risk profile. (2) AT: with (2 points) or without (4 points) high-risk profile. (3) Microvascular involvement: suspected (2 points) or established (5 points). (4) Pregnancy morbidity: three or more consecutive prefetal (<10 weeks) and/or early (10–16 weeks) fetal deaths, or ≥1 fetal death (16–34 weeks) alone (1 point); preeclampsia with severe features or placental insufficiency with severe features (<34 weeks) with or without fetal death (3 points); preeclampsia with severe features and placental insufficiency with severe features (<34 weeks) with or without fetal death (4 points). (5) Cardiac valve: thickening (2 points) or vegetations (4 points). (6) Thrombocytopenia (platelets < 100 × 10^9^/L, 2 points).
Laboratory Criteria	(1) LAC: present in plasma, on two or more occasions at least 12 weeks apart. (2) aCL: IgG and/or IgM present in plasma with medium or high titers (>40 units or >99th percentile) on two or more occasions at least 12 weeks apart. (3) β2-GPI-Ab: IgG and/or IgM present in plasma with titers >99th percentile on two or more occasions at least 12 weeks apart.	(1) LAC: one-time positivity (1 point) or persistent positivity (≥2 positive results, at least 12 weeks apart, 5 points); (2) β2-GPI-Ab/aCL: moderate */high ** β2-GPI-Ab and/or aCL IgM positivity (1 point); moderate * β2-GPI-Ab and/or aCL IgG positivity (4 points); high ** β2-GPI-Ab and/or aCL IgG positivity (5 points); high ** β2-GPI-Ab and aCL positivity (7 points).

Abbreviations: β2-GPI-Ab, anti-β2-glycoprotein I antibodies; aCL, anti-cardiolipin antibodies; AT, arterial thrombosis; EULAR, European League Against Rheumatism; LAC, lupus anticoagulant; VTE, venous thromboembolism. * Moderate β2-GPI-Ab/aCL titers: 40–79 units. ** High β2-GPI-Ab/aCL titers: ≥80 units.
